# Nephrotoxicity in a Patient With Inadequate Pain Control: Potential Role of Pharmacogenetic Testing for Cytochrome P450 2D6 and Apolipoprotein L1

**DOI:** 10.3389/fphar.2019.01511

**Published:** 2020-01-08

**Authors:** Emma M. Tillman, Todd C. Skaar, Michael T. Eadon

**Affiliations:** Indiana University Department of Medicine, Indianapolis, IN, United States

**Keywords:** pharmacogenomic testing, opioid, genetic testing, renal insufficiency, substance-related disorders

## Abstract

A case is presented which demonstrates the perils of opioid inefficacy and how pharmacogenomic testing may have prevented nonsteroidal anti-inflammatory drug (NSAID)-induced nephrotoxicity and progression to chronic kidney disease (CKD). A 62 year-old female with back pain was treated with tramadol and hydrocodone; however, neither proved effective. Consequently, to control her pain, she resorted to cocaine, marijuana, and high dose nonsteroidal anti-inflammatory drugs (NSAIDs). She eventually developed CKD. To identify CKD contributors, she underwent genotyping for Apolipoprotein L1 (*APOL1*), a known risk factor of CKD, as well as relevant pharmacogenomic genes. Her *APOL1* genotype was *G1(GM)/*G1(GM), placing her at increased risk of CKD progression. Her *CYP2D6* genotype was *5/*17, consistent with intermediate metabolism, making opioid drugs reliant on CYP2D6 activation, such as tramadol and hydrocodone, relatively ineffective in this patient. Thus, this patient was at genetic risk for CKD and reduced opioid efficacy. We conclude that this genetic combination likely contributed to opioid inefficacy and the eventual progression to CKD.

## Background

A previous case report demonstrated that not knowing a patient's *CYP2D6* ultra-rapid metabolizer resulted in opioid toxicity and neonatal death (Koren et al., 2006). This was key evidence that resulted in changes in clinical recommendations focused on avoiding opioid toxicity in nursing mothers. Here we present a case illustrating the clinical implications of the extreme opposite effect, not knowing either a patient's *CYP2D6* intermediate metabolizer status resulting in opioid inefficacy or her *APOL1* genetic risk for chronic kidney disease (CKD), and the consequential inability to personalize therapies that likely caused the progression to irreversible chronic kidney disease (CKD). Together with the substantial evidence base indicating the role of CYP2D6 metabolism in opioid effectiveness and *APOL1* and CKD, we believe this report will provide a critical case that further supports the clinical utility of *CYP2D6* and *APOL1* genotyping to guide pain management therapy.

## Case Presentation

A 62 year-old African American woman with a history of chronic osteoarthritic lower back pain presented for evaluation of CKD. On presentation, her creatinine had gradually increased from 1.0 to 1.9 mg/dl over five years, but her blood pressure was well controlled and she was not proteinuric. In addition, her renal ultrasound, urine microscopy, serum protein electrophoresis, and other work-up did not reveal any likely causes of the CKD.

**Figure 1:**
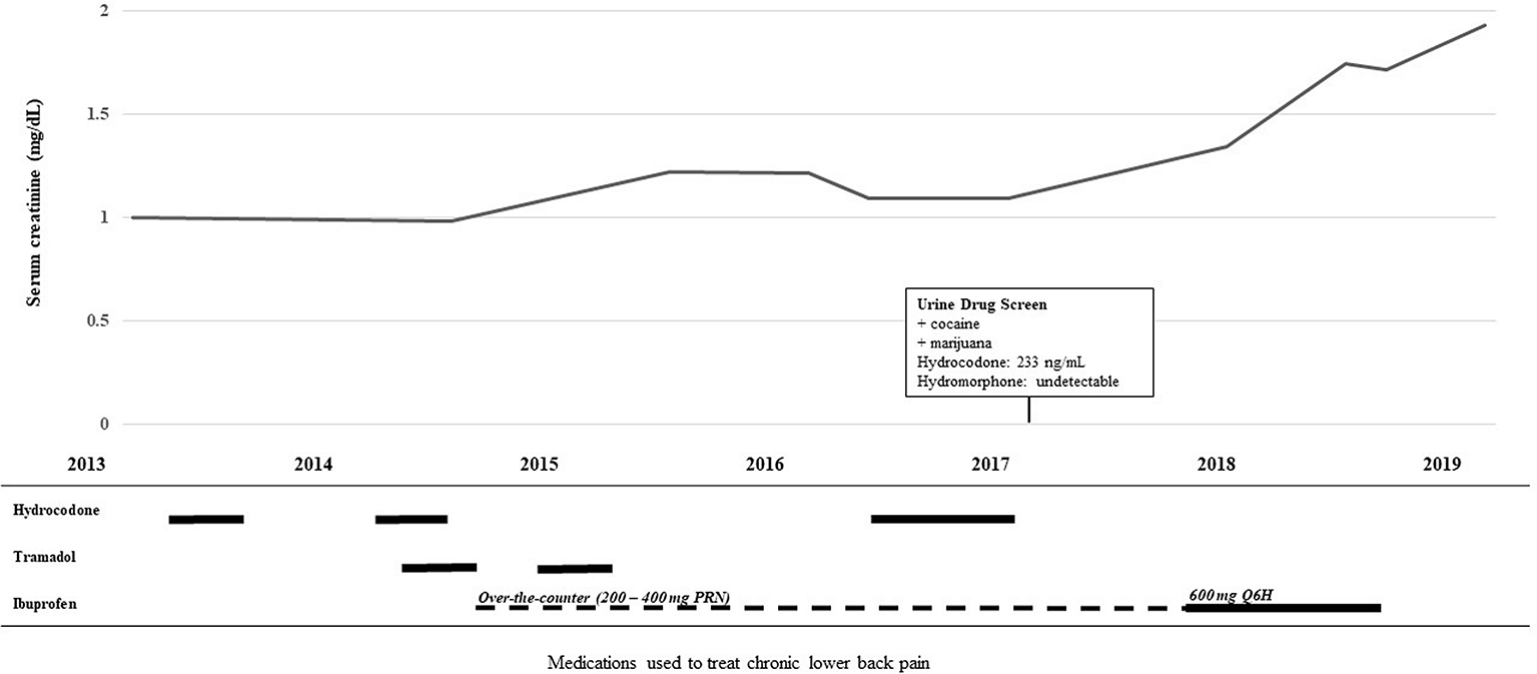
Timeline of medication use and decline in renal function.The top line graph illustrates the rise in serum creatinine over time. The lower histogram illustrates the use of the analgesics hydrocodone, tramadol, and ibuprofen over the same time course. Solid lines depict the patient taking the drug for a consistent period of time and the broken line depicts the *pro re nata* (PRN) “as needed” use.

She had lower back pain which was alternatingly treated with either tramadol 50 mg every 8 h or hydrocodone/acetaminophen 5–325 mg every 6 h as needed, but neither provided symptomatic pain relief. She eventually tested positive on a urine drug screen and admitted to using marijuana and cocaine to alleviate the back pain since the tramadol and hydrocodone were ineffective. Per the positive drug screen policy of the underserved county health clinic, her physicians declined to prescribe further opioids. Instead, her providers prescribed daily high dose of ibuprofen. She had also been taking lower doses of over-the-counter nonsteroidal anti-inflammatory drugs (NSAIDs) on and off for several years (**Figure 1**).

Without any other clear risk factors, the etiology of her CKD was linked to her chronic NSAID use. To identify CKD contributors, she underwent genotyping for Apolipoprotein L1 (*APOL1*), a known risk factor of CKD frequently found in the African American population (Parsa et al., 2013), and cytochrome P450 2D6 (*CYP2D6*), the hepatic enzyme that metabolizes and activates tramadol and hydrocodone. Her *APOL1* genotype was *G1(GM)/*G1(GM), which is a single nucleotide polymorphism that alters *APOL1* placing her at increased risk of CKD progression by** (Freedman, 2013; Freedman et al., 2018). The patient's family history was significant, as her mother progressed to end-stage renal disease. Her *CYP2D6* genotype was *5/*17, a gene deletion which is reported to occur in 2–11% of people and is consistent with intermediate metabolism making tramadol and hydrocodone less effective (Swen et al., 2011; Crews et al., 2014; Linares et al., 2015). A retrospective look at the urine drug screen noted the absence of any hydromorphone, the more active metabolite of hydrocodone, even though hydrocodone was detected (233 ng/ml). Her *CYP2C9* genotype was *1/*8, consistent with reduced CYP2C9 metabolism, the enzyme known to metabolize NSAIDs (Martin et al., 2001).

In addition to her chronic pain, she also had depression which was treated with fluoxetine, which is a CYP2D6 inhibitor that likely reduced any residual CYP2D6 activity. Her fluoxetine dose was slowly titrated from 20 mg to 60 mg daily between 2013 and 2018, but reduced and then stopped after reaching the 60 mg daily dose due to side effects of agitation and lightheadedness. Collectively, these results indicate that the likely cause of the CKD was the high dose of NSAIDS given as a consequence of the ineffectiveness of the opioids in this patient that could not adequately activate the tramadol or hydrocodone. The patient described in this case report has given her written informed consent for genetic testing and to have her medical information published in this manuscript.

## Discussion

This patient's genotype predicts a CYP2D6 intermediate metabolizer phenotype which has been shown to occur in ~13% of African Americans; thus, pharmacogenomic testing would have predicted substantially lower active metabolites of tramadol and hydrocodone and consequently, inefficacy (Swen et al., 2011; Crews et al., 2014; Linares et al., 2015). In addition, she was also taking fluoxetine to treat her depression, a common co-morbidity with chronic pain, which would have reduced further her endogenous CYP2D6 activity. Although both tramadol and hydrocodone do have some direct activity on the mu receptors (Lelas et al., 1999), their metabolites (M1 and hydromorphone) are 200 and 20 times stronger than the parent drugs respectively, providing a major part of the analgesic effect of these drugs; thus, tramadol and hydrocodone are much less active in CYP2D6 reduced and poor metabolizers. Consistent with the known CYP2D6 mediated metabolism of hydrocodone and the patient's CYP2D6 status, this patient's urine drug screen did not detect any hydromorphone, the active metabolite of hydrocodone, despite the presence of 233 ng/ml of hydrocodone (lower limit of detection 25 ng/ml for hydrocodone and hydromorphone). The metabolic ratio of hydromorphone to hydrocodone in the general population is 0.161 and her levels are consistent with reduced CYP2D6 metabolism (Barakat et al., 2012). Since this result was obtained retrospectively after her *CYP2D6* genotyping and her termination of any opioid prescription per the health care system's policy, we were not able to prescribe any additional opioids or obtain blood for analysis while she was prescribed these medications. In patients without the ability to activate tramadol and hydrocodone, other opioids that are not dependent on CYP2D6 metabolism, such as transdermal fentanyl or hydromorphone, would likely have provided greater pain relief. Without *a priori* knowledge of opioid efficacy, the patient's clinicians were unable to select an appropriate drug to control her pain, precipitating irreversible CKD.

Cases of rare adverse drug events can demonstrate the utility of pharmacogenetics. The report of morphine poisoning in a breast-fed infant of a mother taking codeine is another case of a CYP2D6 mediated adverse drug event (Koren et al., 2006). That case sparked a scientific investigation and substantial attention that culminated in changes to codeine prescribing in nursing mothers. This case demonstrates once again, the potential value for *CYP2D6* genotyping for guiding opioid therapies, but also illustrates that screening for inefficacy can be as important as toxicity.

The *G1/*G1 APOL1 genotype, common observed in the African American population, is known to increase the severity of progression in many forms of CKD, including hypertensive CKD, lupus-related kidney disease, focal segmental glomerulosclerosis, sickle cell nephropathy, and HIV-associated kidney disease (Freedman, 2013; Freedman et al., 2018). While there are currently no guidelines to recommend withholding NSAIDs in patients with a positive *APOL1* genotype, high-dose NSAID use has been shown to significantly increase the risk of accelerated CKD (Nderitu et al., 2013). At the time our patient was prescribed high dose ibuprofen, her *APOL1* genotype was unknown and she was not educated regarding the use of over-the-counter NSAIDs. Patients at higher risk for CKD should be counseled on environmental factors such as NSAIDs, cocaine, and other nephrotoxins that can be avoided. Finally, the patient carried genetic variants that reduced CYP2C9 activity, and an enzyme that is involved in the metabolism of NSAIDs. It is not known whether reduced CYP2C9 metabolizers who take NSAIDs have an increased risk of kidney disease, but systemic exposure may be increased. Given our patient's genetic predisposition, the extended and high-dose NSAID exposure in this patient, with a contribution from her cocaine use, were the likely causes of her CKD progression. This case provides additional support that genetic testing holds utility for screening both drug-gene interactions and genetic risk factors. The use of pharmacogenomic-guided opioid therapy may serve as a valuable tool in selecting appropriate pain regimens, reducing adverse events, and objectively reinforcing inefficacy.

## Data Availability Statement

The datasets for this manuscript are not publicly available because they may contain identifying participant information. Anonymized data such as phenotype/genotype and case/control classification can be requested. Requests to access the datasets should include a letter indicating the intended use and appropriate approval by your institution. This should be directed to the corresponding author.

## Author Contributions

All authors (ET, TS, ME) had substantial contributions to the acquisition, analysis, and interpretation of this case, drafting and critically revising the manuscript, and have agreed to the final manuscript.

## Conflict of Interest

The authors declare that the research was conducted in the absence of any commercial or financial relationships that could be construed as a potential conflict of interest.
